# Role of hepatitis B virus non-structural protein HBx on HBV replication, interferon signaling, and hepatocarcinogenesis

**DOI:** 10.3389/fmicb.2023.1322892

**Published:** 2023-12-21

**Authors:** Fei Wang, Hongxiao Song, Fengchao Xu, Jing Xu, Le Wang, Fan Yang, Yujia Zhu, Guangyun Tan

**Affiliations:** ^1^Department of Hepatology, Center for Pathogen Biology and Infectious Diseases, Institute of Translational Medicine, The First Hospital of Jilin University, Changchun, Jilin, China; ^2^Health Examination Center, The First Hospital of Jilin University, Changchun, China; ^3^Department of Hepatology, The First Hospital of Jilin University, Changchun, China; ^4^Department of Anesthesiology, The First Hospital of Jilin University, Changchun, Jilin, China

**Keywords:** HBx, HBV, interferon (IFN), HCC, ISGs (IFN-stimulated genes)

## Abstract

Hepatitis B, a global health concern caused by the hepatitis B virus (HBV), infects nearly 2 billion individuals worldwide, as reported by the World Health Organization (WHO). HBV, a hepatotropic DNA virus, predominantly targets and replicates within hepatocytes. Those carrying the virus are at increased risk of liver cirrhosis and hepatocellular carcinoma, resulting in nearly 900,000 fatalities annually. The HBV X protein (HBx), encoded by the virus’s open reading frame x, plays a key role in its virulence. This protein is integral to viral replication, immune modulation, and liver cancer progression. Despite its significance, the precise molecular mechanisms underlying HBx remain elusive. This review investigates the HBx protein’s roles in HBV replication, interferon signaling regulation, and hepatocellular carcinoma progression. By understanding the complex interactions between the virus and its host mediated by HBx, we aim to establish a solid foundation for future research and the development of HBx-targeted therapeutics.

## Introduction

Hepatitis B virus (HBV) has infected 2 billion people globally, with 240 million suffering chronic infections, contributing significantly to morbidity and mortality in endemic regions (Ishida et al., 2018). Moreover, chronic HBV infections increase the risk of liver diseases such as liver fibrosis, cirrhosis, and hepatocellular carcinoma (HCC) (Gallay et al., 2019). HBV is an enveloped DNA virus of the Hepadnaviridae family (Seitz et al., 2007). To date, only humans and orangutans are known to be susceptible to HBV, leading to hepatitis B disease. The fully-formed HBV, also called Dane particles, has a pivotal phase in its life cycle marked by covalently closed circular DNA (cccDNA) formation. This cccDNA acts as the template for generating HBV RNAs (Huang et al., 2018). HBV particles enter cells by binding to the sodium taurocholate cotransport polypeptide (NTCP) receptor. Upon endosomal maturation, the viral envelope fuses with the endosomal membrane, releasing the nucleocapsid into the cytoplasm (Tan et al., 2018). The translocation of the nucleocapsid to the nucleus releases loosely looped DNA. With the help of the cellular replication machinery, some of the double-stranded DNA is converted to cccDNA, which further interacts with histones H3, H4, and non-histone proteins to form viral microchromosomes (Yuen et al., 2018).

In addition, cccDNA, as a transcription template, encodes four major viral RNAs: pre-genomic RNA (pgRNA, 3.5 kb), preS1 HBs RNA (2.4 kb), preS2/S HBs RNA (2.1 kb), and HBV x RNA (HBx, 0.7 kb). These viral mRNAs then migrate to the cytoplasm after nuclear synthesis, acting as templates for the synthesis of viral proteins or negative strand DNA (reverse transcription). The pgRNA interacts with the core protein to form a new nucleocapsid. Virus particles, with a diameter of 42 nm, consist of two primary components: the outer shell (or envelope) and the core. The virus’s outer shell, or envelope, comprises hepatitis B surface antigen (HBsAg) and phospholipids from infected cells, encasing the icosahedral nucleocapsid. The nucleocapsid contains 240 copies of the HBV core protein and a segment of the double-stranded HBV genome. The HBV genome has a total length of about 3.2 kb in the form of a double-stranded circular DNA. Its genome is composed of four partially overlapping open reading frames (ORFs).

HBV mRNA encodes seven proteins according to the position of the starting codon in the RNA transcript. These mRNAs and proteins encoded by them are: Prec mRNA (3.5 kb), which synthesizes the HBV e antigen (HBeAg); pgRNA (3.5 kb), responsible for the HBV c antigen (HBcAg) and polymerase (PoL); PreS2/S mRNA (2.4 kb) for the large S protein; pre-S1 mRNA (2.1 kb) for small and medium surface proteins (MS and HBsAg); and HBx mRNA (0.7 kb), which forms HBV x antigen (HBxAg) (Tan et al., 2018). The X protein of HBV is crucial for the virus’s replication and is associated with the onset of liver cancer. Conversely, the S protein forms the viral envelope and plays a pivotal role in facilitating the entry of the virus into the cell. Once the viral proteins reach a specific concentration in the cytoplasm, both pgRNA and the Pol protein undergo selective packaging into HBcAg, forming an immature nucleocapsid. Subsequently, under the catalysis of the Pol reverse transcriptase (RT) domain, this undergoes reverse transcription into negative-strand DNA. This negative strand then acts as a template for synthesizing a variable length of positive-strand HBV DNA, ultimately generating the HBV genome. The mature HBV capsid follows one of two paths: it is either enveloped by the HBsAg and excreted from the liver cells or is recycled back to the nucleus to augment the cccDNA pool. Eliminating cccDNA from infected liver cells poses a formidable challenge in HBV biology (Tong and Revill, 2016). In recent advancements, studies have employed the CRISPR/Cas9 technology combined with DNA imaging and the HBV microloop system. This approach has enabled the construction of a cellular model wherein cccDNA molecules are labeled with fluorescent proteins, allowing for high-sensitivity, high-specificity real-time tracking of cccDNA at the single-cell level (Ding et al., 2023).

The HBx protein, consisting of 154 amino acids and a molecular weight of 17 kDa, is highly conserved among all mammalian liver viruses (Peng et al., 2005). Structurally, the HBx protein possesses an N-terminal negative regulatory domain. On the other hand, the C-terminal domain, through its anti-activation or co-activation properties, interferes with the host cell’s signal transduction pathway, promoting HBV replication (Song et al., 2021).HBx is a multifunctional, non-structural protein. Within the virus’s life cycle, the HBx protein, post-infection, plays a crucial role in initiating and maintaining HBV replication (Lucifora et al., 2011). Research has shown that mutations in HBx significantly reduce the replication efficiency of its mutants, both *in vitro* and *in vivo* (Zhang et al., 2018). Being a complex trans-activator, HBx has multiple roles in various HBV-associated liver cancers (Kremsdorf et al., 2006).

Interferons (IFNs), a superfamily of cytokines known for their antiviral properties, are crucial in the body’s response to viral infections, including HBV (Lindenmann et al., 1957). It has been observed that IFNs not only inhibit virus replication but also play roles in cell proliferation, apoptosis, inflammation, and adaptive immunity (Stark et al., 1998). IFNs, a broad-spectrum antiviral agent, do not directly neutralize the virus. Instead, they act on cell surface receptors, inducing cells to produce antiviral proteins that inhibit HBV replication (Randall and Goodbourn, 2008). At the same time, IFNs can also enhance the activity of natural killer cells (NK cells), macrophages, and T lymphocytes, thus regulating immunity and enhancing antiviral defenses (Sadler and Williams, 2008). In response to microbial infections, host cells release IFNs. Human interferons are classified into three main types based on their receptor affinities: type I, type II, and type III. Type I interferons, including IFN-α, IFN-β, IFN-ε, IFN-κ, and IFN-ω, are approved as a first-line treatment for chronic HBV. In addition to direct antiviral effects, IFN treatment enhances the body’s immune response against HBV. However, the efficacy of IFN treatment is limited, and its significant side effects hinder broader clinical application. This review aims to elucidate how the HBx protein influences HBV replication, hepatocarcinogenesis, and IFN signaling.

### Effect of HBx on HBV replication

The HBx protein, a non-structural component of the HBV, plays pivotal roles in several aspects of the virus’s lifecycle, especially in regulating HBV replication and RNA transcription. HBx significantly impacts HBV replication by inhibiting the RIG-I-mediated innate immune pathway, particularly through targeting the mitochondrial antiviral signal protein (MAVS) (Wang et al., 2020). HBx is instrumental in initiating and maintaining the transcription from the cccDNA template. This was observed in a cell line designed to stably express HBx when exposed to a specific concentration of tetracycline (TET). In this model, the introduction of TET resulted in sustained HBeAg secretion. However, cessation of TET led to a decline in levels of pgRNA, HBV DNA, HBeAg, and HBsAg. Fascinatingly, reintroducing HBx resulted in the resumption of viral replication, highlighting the ongoing need for HBx in HBV transcription and replication (Lucifora et al., 2011). Comprehensive studies using HBV plasmid replication techniques and the tail vein injection model have clarified HBx’s mechanisms and functions within the host. Notably, these studies confirmed a significant presence of HBx in the livers of mice afflicted with acute hepatitis (Keasler et al., 2007).

When HBV infects hepatocytes, the LS protein on the surface of virus particles is recognized and bound by the sodium taurocholate cotransporter peptide (NTCP) receptor on the surface of hepatocytes and enters the cells through endocytosis. The nucleocapsid is released from the cytoplasm. Under the action of the nuclear localization signal of the core protein, the nucleocapsid enters the nucleus, releases relaxed-circular (RC DNA), and finally forms cccDNA. Current therapeutic strategies face challenges in eradicating cccDNA, a key contributor to chronic hepatitis B infection. Acting as a replication intermediary, cccDNA resides in the host liver nucleus as microchromosomes and serves as a template for HBV replication. The HBx protein, crucial for HBV replication and associated carcinogenesis, can bind to cccDNA microchromosomes, playing a pivotal role in initiating and maintaining cccDNA transcription (Zhang et al., 2020). Studies have demonstrated that HBx interacts with cccDNA microchromosomes and various histone-modifying enzymes, influencing HBV transcriptional regulation (Chong et al., 2020).

Studies have clarified how NQO1 gene modulation and dicoumarin treatment affect HBx protein’s recruitment to cccDNA. Specifically, the knockout of the NQO1 gene and the administration of dicoumarin reduce HBx’s association with cccDNA, leading to the attenuated transcriptional activity of cccDNA. This observed inhibition is potentially attributable to changes in the establishment of chromatin states. Importantly, upon deletion of HBx, the antiviral efficacy conferred by NQO1 gene disruption and dicoumarin treatment against HBV infection is ablated. This underscores the pivotal role of the NQO1 gene in regulating the stability and function of the HBx protein during HBV replication (Cheng et al., 2021).

Several studies highlight ethanol’s modulatory effects on cccDNA dynamics, with IFN-α2b serving as an inhibitor against ethanol-mediated cccDNA promotion. Ethanol promotes cccDNA expression through HBx-mediated activation of MSL2. Conversely, IFN- α2b mitigates cccDNA concentrations by inhibiting MSL2, thereby disrupting the positive feedback loop constituted by HBx/MSL2/cccDNA/HBV/HBx, which otherwise amplifies ethanol-induced HBV replication (Liu et al., 2020). Additionally, data substantiate the role of HMGA1 as a salient up regulator of HBV activity. Notably, HBx-driven induction of HMGA1 by HBV highlights a key aspect of viral activity regulation. This reciprocal upregulation between HMGA1 and HBV, facilitated by HBx, is a prospective therapeutic target for hepatitis B patients (Shen et al., 2022).

**Figure 1:**
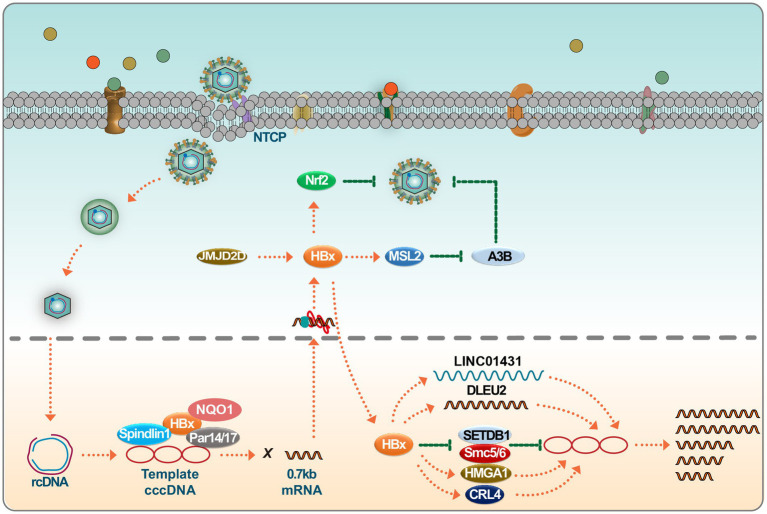
HBx regulates HBV replication. Upon infection, the HBV genome undergoes transcription to produce 0.7 kb of HBx mRNA. Several host factors, including Spindlin1, NQO1, and Par14/17, interact with HBx, subsequently promoting cccDNA transcription. HBx interacts with long noncoding RNAs (lncRNAs) such as LINC01431 and DLEU2, facilitating HBV replication. HBx also engages with known cccDNA inhibitors, including SMC5/6 and SETDB1, neutralizing their inhibitory effects on the viral genome. HBx binds to positive regulators like CRL4 and HMGA1 to augment HBV transcription, furthering the viral life cycle. Interestingly, the host can hijack HBx to stabilize Nrf2, thereby impairing HBV replication. Please see the text for details.

Studies have demonstrated that the highly conserved N-terminal of HBx interacts with Tudor3 of Spindlin1, influencing biological function, and this interaction enhances the gene expression of chromatin cccDNA. This conformational switch in HBx can coordinate with other binding factors like DDB1, ultimately promoting HBV transcription of its chromatin genome (Ducroux et al., 2014; Liu et al., 2023). In addition, parvulin 14 and parvulin 17 bind to HBx and cccDNA and upregulate the replication of the HBV from cccDNA to viral particles in an HBx-dependent manner (Saeed et al., 2019). Recent studies have shown that HBV infection can trigger the expression of JMJD2D. JMJD2D interacts with the HBx virus protein to demethylate H3K9me3 on HBV cccDNA, thereby hindering p53 from binding to HBV cccDNA (Kong et al., 2023). This process facilitates HBV transcription and replication. The HBx virus protein is crucial in HBV replication, as HBV manipulates the Cullin4-DDB1-RING (CRL4) ubiquitin ligase for viral purposes through the oncoprotein HBx (Zeng et al., 2023). In recent years, more and more studies have been conducted on miRNA and its sponge molecule, lncRNA. As a new type of long intergenic noncoding RNAs (lincRNAs), LINC01431 has been reported to bind to protein arginine methyltransferase 1 (PRMT1), which in turn mediates the inhibition of LINC01431 on HBV replication (Sun et al., 2022). Recent research indicates that HBx binds to the promoter region, enhancing transcription and inducing the accumulation of deleted lymphocytic leukemia 2 (DLEU2) in infected hepatocytes. The co-recruitment of HBx and DLEU2 on cccDNA replaces enhancer of zeste 2 polycomb repressive complex 2 Subunit (EZH2) in viral chromatin, thus promoting transcription and viral replication (Salerno et al., 2020). While IFNs reduce the cccDNA load and amplify the interferon-stimulated gene, their antiviral effect decreases post-treatment, leading to a decline in the structural maintenance of chromosome 5/6 (SMC5/6) complex levels. Treatment methods that remove all HBV transcripts, including HBx, enhance epigenetic inhibition of HBV microchromosomes. However, a strategy to shield human hepatocytes from reinfection is essential to maintain cccDNA silence (Allweiss et al., 2022). Studies have shown that cellular mechanisms involving SETDB1-mediated H3K9me3 and HP1 induce transcriptional silencing of HBV cccDNA after HBV infection by regulating chromatin structure, whereas HBx can alleviate this inhibition and allow the establishment of active chromatin (Rivière et al., 2015). Recently, studies have shown that HBx degrades the SMC5/6 complex, which is crucial for HBV replication (Murphy et al., 2016). Studies show that HBx facilitates the ubiquitination of SMC5/6 by CRL4 (HBx) E3 ligase, leading to its degradation by the proteasome (Hou et al., 2019). At the same time, the degradation of SMC5/6 complexes is pivotal in DNA damage accumulation and HBx-driven tumorigenesis (Sekiba et al., 2022) (Figure 1).

In conclusion, although fully eradicating cccDNA from infected livers is challenging, recent studies highlight potential impacts on cccDNA transcription by targeting HBx with inhibitors, antibodies, or other therapies. This knowledge paves the way for developing enhanced treatments targeting chronic HBV infection. Notably, HBx has been observed to be co-opted by the host to stabilize Nrf2, subsequently inhibiting HBV replication (Ariffianto et al., 2023). Thus, the potentially beneficial effects of HBx within the host should be factored into HBx-centric research and therapeutic development.

### HBx promotes hepatocarcinogenesis

HBx has been reported to be involved in the pathogenesis of HCC. Studies suggest that HBx’s elevation of MSL2 modulates HBV cccDNA in HCC cells, potentially contributing to the onset of HCC (Gao et al., 2017). Additionally, HBx stabilizes the AIB1 protein, and this synergistic interaction with AIB1 enhances the invasiveness of HCC cells. This finding underscores the significance of the interaction between HBx and AIB1 in the progression of HBV-associated HCC (Liu et al., 2012). Furthermore, HBx appears to downregulate lncRNA-DREH, which, in turn, binds to the intermediate filament protein vimentin. This binding event inhibits vimentin expression, leading to changes in the typical cytoskeletal structure and potentially acting as a barrier to tumor metastasis (Huang et al., 2013). Notably, in the context of liver cancer arising from chronic HBV infection, HBx has been identified as a factor that induces anoikis resistance in liver cancer cells. This is achieved by its upregulation of PAK1, which further promotes the growth of invasive xenografts in murine models (Xu et al., 2012).

The role of HBx in HCC progression is complex, affecting various signaling pathways to influence metastasis and invasion. One mechanism through which HBx mediates HCC is via the Hippo signaling pathway. Specifically, YES-associated protein (YAP), an established oncogene, has been linked to HBx-mediated HCC (Zhang et al., 2012). Another study highlighted that HBx interacts with MYH9, inducing its expression through the GSK3β-β-catenin/c-jun signaling pathway. Consequently, targeting MYH9 might be a potential therapeutic approach to impede tumorigenesis and epithelial-mesenchymal transition (EMT) (Lin et al., 2020).

In tumor biology, NF-κB serves as a central transcriptional driver. Evidence suggests that HBx-mediated activation of the NF-κB signaling pathway augments the expression of pro-metastatic genes such as VEGF and MMPs, thereby promoting HCC cell metastasis and invasion. Inhibitors like PDTC, which target the NF-κB pathway, have been shown to block this process (Liu et al., 2010). In addition to these signaling pathways, another molecular mechanism has been identified: HBx appears to stimulate FoxM1 expression via the ERK/CREB pathway, which may contribute to liver cancer cell invasiveness and metastasis (Xia et al., 2012). Furthermore, the potential therapeutic properties of natural polyphenols have garnered attention in recent research. For example, resveratrol, a natural polyphenol, has been shown to exert protective effects against HBV-associated HCC. Resveratrol’s mechanism of action includes the inhibition of LXRα, leading to the down-regulation of lipogenic genes such as SREBP1-C and PPARγ. Such modulatory effects suggest that HBx-mediated liver cancer progression could, in certain contexts, be attenuated by resveratrol (Lin et al., 2012; Ning et al., 2022).

LncRNAs have emerged as pivotal regulators in innate immunity, displaying dual roles. They can enhance antiviral responses by modulating mechanisms like receptor activities and key molecular dynamics within signaling pathways. Conversely, some lncRNAs act as oncogenic agents, mediating chronic inflammation, malignant cell recognition, and invasion, thus facilitating tumor progression (Lei et al., 2022). LncRNAs can also exert indirect control over miRNA expression via interactions with proteins, further influencing tumor growth. Research has indicated that miR-3188 knockout can directly restrain ZHX2, exhibiting antitumor properties. However, HBx can counteract this by stimulating CREB-mediated activation of miR-3188 and the Notch signaling pathway via ZHX2 inhibition, underlining its significance in HBV-related liver cancer (Zhou et al., 2017). Another study highlighted the role of HBx in augmenting TRERNA1 expression, which subsequently reduces the sensitivity of HCC cells to sorafenib by triggering the RAS/Raf/MEK/ERK pathway. This research also unveiled a novel regulatory framework whereby HCC progression is mediated through the TRERNA1/miR-22-3p/NRAS axis, suggesting TRERNA1 as a potential tumor biomarker and therapeutic target (Song et al., 2021).

Survivin, a unique apoptosis suppressor protein, is exclusively expressed in tumor and embryonic tissues. This protein is complexly linked with tumor cell differentiation, proliferation, invasion, and metastasis. Studies suggest that HBx, alongside miR-520b and HBxIP, promotes liver cancer progression by influencing Survivin (Zhang et al., 2014). Moreover, HBx has been shown to suppress miR-187-5p expression through the E2F1/FoxP3 axis, consequently enhancing HCC cell growth, migration, and invasion (Deng et al., 2023). Studies have illuminated the role of HBV-LINE1 in acting as a molecular sink for miR-122, leading to its depletion. This results in the activation of the β-catenin signaling pathway, reduced E-cadherin expression, aberrant hepatocyte mitosis, and liver damage in mice (Liang et al., 2016). In addition to the significant roles of lncRNA and miRNA in the development and progression of liver cancer, the circular RNA cFAM210A, degraded by the HBx, plays a protective role against liver cancer by inhibiting YBX1-mediated trans-activation (Yu et al., 2023).

HBx has been demonstrated to interact with the farnesoid X receptor (FXR) and can serve as an FXR co-activator. Interestingly, a C-terminal truncated HBx variant commonly detected in clinical HCC cannot trans-activate FXR. The trans-activation of FXR by the full-length HBx might be a defensive mechanism against HCC progression (Niu et al., 2017). Other studies have also confirmed the significance of the HBx C-terminal region. Truncation of its last 24 amino acids was found to amplify the invasive and metastatic capabilities of HCC cells. This effect was mediated through the activation of MMP10 via c-jun (Sze et al., 2013).

Moreover, increasing evidence points towards the upregulation of DDX17 by HBx. Elevated DDX17 levels enhance HBV replication and transcription, an effect attributed to ZWINT upregulation. Additionally, DDX17 has been linked to the metastasis of liver cancer under the influence of HBx (Dong et al., 2022). HBx also promotes hepatocarcinogenesis by directly associating with CDK2. This interaction induces the phosphorylation of UHRF2 at serine 643, inhibiting UHRF2 ubiquitination (Cheng et al., 2021). Significantly, HBx stimulates alpha-fetoprotein (AFP) expression, leading to hepatocyte reprogramming and impacting liver cancer development. AFP is key to initiating hepatoma progenitor/stem cells, so it is a promising therapeutic target against HBV-induced HCC (Zhu et al., 2017).

Another pivotal epigenetic factor in HBV-associated tumorigenesis is WDR5. Data indicates that the HBx-WDR5-H3K4ME3 axis could be targeted for therapeutic interventions in HBV-induced liver pathogenesis (Gao et al., 2020). Concurrently, HBx has been shown to disrupt the CRL4 (WDR70)-mediated DNA end excision process, culminating in homologous recombination (HR) deficiency in HBV-infected hepatocytes. This damaged double-strand break (DSB) repair accelerates genomic instability, further driving the progression of liver cancer (Ren et al., 2019).

**Figure 2:**
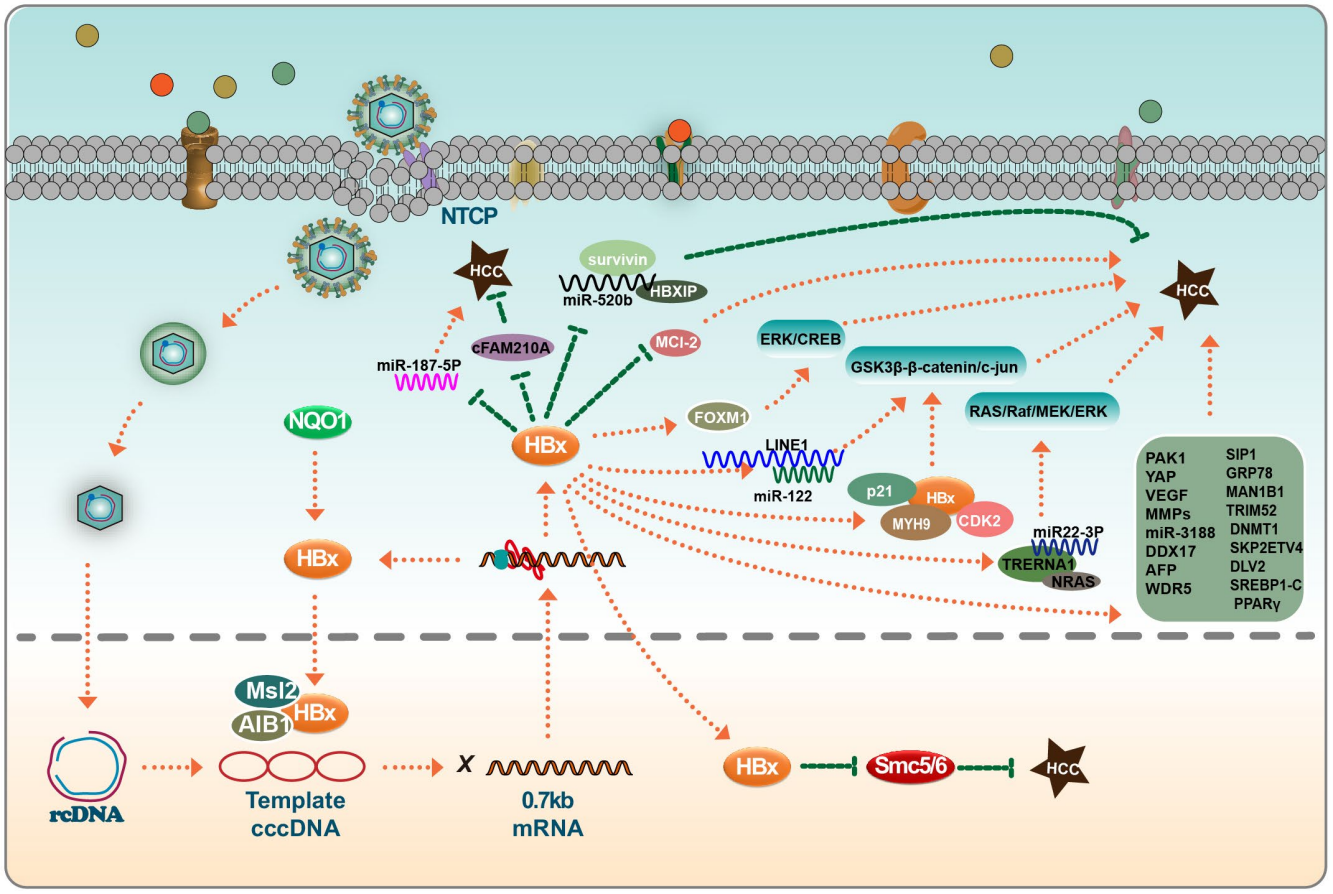
HBx viral proteins play important roles in hepatocellular carcinoma. In the nucleus, HBx binds to MSL2 and AIB1 and regulates the synthesis of HBV cccDNA in hepatocellular carcinoma cells; HBx also inhibits the tumor-suppressive effects of SMC5/6. In the cytoplasm, HBx has a bidirectional effect on miRNAs, which can target some miRNAs to promote or inhibit hepatocarcinogenesis, such as miR-520b, miR-187-5P, miR-122, miR22-3P, and miR-3188. Interestingly, these miRNAs do not act independently. The co-factors that play a role in this process include survivin, HBXIP, Mcl-2, FOXM1, p21, MYH9, and CDK2, among others. HBx also directly affects the expression levels of a group of genes (MMPs et al.), which ultimately induces EMT in hepatocytes and tumor cell invasion. Please see the text for details.

HBx-induced upregulation of ETV significantly increases DVL2 expression, thereby enhancing HCC cell invasion and metastasis. In particular, the discovery of the ETV4-DVL2-β-catenin axis in HBV-associated HCC offers new therapeutic avenues for aggressive HCC (Zheng et al., 2022). HBx has also been found to enhance the expression of SMAD interacting protein 1 (SIP1), recruiting it to the E-cadherin promoter and suppressing E-cadherin transcription. Recent studies identify ARRB1 as a key modulator in HBV-associated HCC, highlighting its therapeutic potential due to its role in autophagy and the CDKN1B-CDK2-CCNE1-E2F1 signaling pathway (Lei et al., 2021). Another factor, histone deacetylase 1, is involved in the formation of an inhibitory complex, suggesting its role in liver cell EMT and tumor invasion (Ye et al., 2019). Moreover, the role of the GRP78 protein, a binding entity localized in the endoplasmic reticulum, has been highlighted. HBx appears to augment GRP78’s stability through TRIM25, amplifying the expression of MAN1B1 to potentially promote tumorigenesis (You et al., 2023). While TRIM25, TRIM26, and TRIM28 have been established as inhibitors of HBV replication, newer research suggests that HBx’s ectopic expression can upregulate TRIM52 in HepG2 cells. Importantly, TRIM52 silencing was found to hinder HepG2.2.15 cell proliferation. Furthermore, TRIM52 has been reported to augment cell proliferation, and HBx may modulate its expression in HBV-associated HCC via the NF-κB signaling pathway (Zhang et al., 2017). The HBx protein has also been linked to enhanced metastasis and chemotherapy resistance in HCC, with evidence suggesting the involvement of SKP2 (Su and Yu, 2019). Epidemiological data indicate a gender disparity in HCC incidence, with males being more affected than females. Interestingly, both male and female p21-HBx transgenic mice are susceptible to HCC. However, male p21-HBsAg transgenic mice displayed an onset of HCC 3 months earlier (Wang et al., 2004). HBx accelerates MCL-1 protein loss through the caspase-3 cascade in oxidative stress conditions, exacerbating HBV-related liver diseases. This insight might shed light on the molecular mechanism of HBV-associated liver cancer (Hu et al., 2011). Prior investigations have also revealed that HBx can transcriptionally modulate DNA methyltransferase DNMT1, leading to hypermethylation in specific regions of tumor suppressor genes and global genomic hypomethylation. Thus, soon after HBV infection, HBx regulates the expression of DNMTs, potentially accelerating the epigenetic progression of liver cancer (Park et al., 2007) (Figure 2).

In summary, HBV stands as a principal contributor to both acute and chronic viral hepatitis, often serving as a precursor to HCC. The HBx protein, crucial in HBV biology, functions both as a transcription factor for HBV genes and a multifaceted trans-activator. Structurally, the distinct domains within HBx, particularly the N-terminal and C-terminal regions, modulate host cell signal transduction pathways (Martin-Vilchez et al., 2011). This modulation disrupts the expression of genes such as MLS2, A1B1, MYH9, FXR, DDX17, and ETV4. Such alterations in gene expression are pivotal in the pathogenesis of HCC. Beyond its influence on gene expression, HBx significantly interacts with specific miRNAs and lncRNAs. This complex network of interactions mediated by HBx culminates in the EMT in hepatocytes, paving the way for tumor invasion and the onset of HCC.

## The interaction of HBx and IFN signal pathway

### Regulation of interferon by HBx

Host cells respond to viral invasion by activating innate immune responses via pattern recognition receptors (PRRs). Primarily, these include Toll-like receptors (TLRs) and RIG-I-like receptors (RLRs) (Wang et al., 2020; You et al., 2022). Activation of these PRR signaling pathways culminates in recruiting various transcription factors, notably IRF3. This cascading effect subsequently triggers the production of type I IFNs, proinflammatory cytokines, and chemokines. These molecules counter viral replication and transmission while also triggering the adaptive immune response (Schneider et al., 2014). Recent findings have underscored the ability of the innate immune system to recognize and respond to HBV, an essential function in controlling HBV infection. In this context, RIG-I has been identified as a key sensor for HBV, pivotal in initiating innate signaling pathways. Importantly, HBV has evolved strategies to modulate host innate immune responses, promoting its survival and proliferation.

IFN is renowned for its broad-spectrum antiviral properties and is a significant alternative therapeutic for HBV infection. IFN primarily exerts antiviral effects by modulating interferon-stimulated genes (ISGs). In the early phases of cell infection, most viruses are detectable by PRRs, which mediate the innate immune response primarily via IFNs. Recent experimental findings have highlighted the capability of HBx to suppress interferon production.

HBx negatively regulates type I IFN production by targeting key signaling components, including RIG I, TRAF3, Cardif, TRIF, Nemo, TBK1, kinase ε (IKKε), and Interferon Regulatory Factor 3 (IRF3) (Jiang and Tang, 2010). The DNA sensor cGAS is vital in the innate immune response following microbial infection, cellular stress, and cancer development. When activated by double-stranded DNA present in the cytoplasm, cGAS synthesizes 2′3’ cyclic GMP-AMP, producing inflammatory cytokines and type I IFN (Pathare et al., 2020). Experimental evidence suggests that HBx interferes with the cGAS/STING signaling pathway. Specifically, HBx induces cGAS ubiquitination and degradation, thus reducing type I IFN production (Schneider et al., 2014; Chen et al., 2022). This interference by HBx aids in creating a favorable environment for HBV replication and survival within the host. HBx has also been reported to modulate the RIG-I signaling pathway. Specifically, HBx mediates the downregulation of MAVS, an essential component of this pathway. This action attenuates the antiviral response, as evidenced by the interference with the ubiquitination process at the Lys136 residue in the MAVS protein to promote MAVS degradation, leading to the inhibition of IFN-β production (Wei et al., 2010). Mutational studies have provided insights into the regulatory dynamics of HBx. Notably, the R87G and I127V mutations in HBx counter its inhibitory role by preventing MAVS degradation, subsequently restoring IFN-β production. Clinical correlations suggest that these mutations are associated with a reduced HBV immune tolerance phase in patients with slow hepatitis B (Chang et al., 2022). Furthermore, genetic variations in HBx also influence the innate immune response to HBV. Functional analyses have shown that the presence of asparagine (N) and glutamate (E) at positions 118 and 119 in the HBx protein exhibit a greater propensity to inhibit RIG-I signaling compared to the lysine (K) and aspartic acid (D) variants, with enhanced interaction affinity with MAVS being observed for the former (Wang et al., 2020). Another study identified that UBXN7, a negative regulator of NF-kB, degradation by HBx promotes HBV replication. This effect is postulated to arise from the maintained activation of IKK-B, which activates the NF-kB signaling pathway and NF-kB-dependent autophagy. Targeting UBXN7 may present a therapeutic approach for HBV-related conditions (Yuan et al., 2022). HBx has also demonstrated an affinity for beta interferon promoter stimulator 1 (IPS-1), a pivotal adaptor protein for IFN-β activation. The binding of HBx to IPS-1 results in decreased IFN-β production, further highlighting the multifaceted approach HBx adopts in impeding the host’s immune response (Kumar et al., 2011).

Type I IFN, particularly IFN α, is a cornerstone of current HBV therapy, though its effectiveness varies among patients. However, clinical outcomes with IFN α therapy are suboptimal, with only 20–40% of patients demonstrating favorable responses (Shen et al., 2018). Clinical data suggests that HBV’s viral genotype significantly influences interferon therapy’s therapeutic outcome. However, the cellular IFN response is even more significant than the viral genotype, demonstrating a higher correlation to therapeutic sensitivity. A major challenge to the therapeutic efficacy of IFN arises from the ability of HBV to subvert and weaken the antiviral function of IFN. Cellular investigations reveal that the HBx protein of HBV interacts with SMC5/6, while MMP9 associates with type I IFN receptor (IFNAR), and Rubicon binds to NEMO. These protein–protein interactions critically impair IFN signal transduction, consequently facilitating HBV infection and persistence. This insight suggests that small molecules disrupting these protein interactions could counter HBV’s suppression of IFN signaling, enhancing treatment efficacy (Tan et al., 2018).

**Figure 3:**
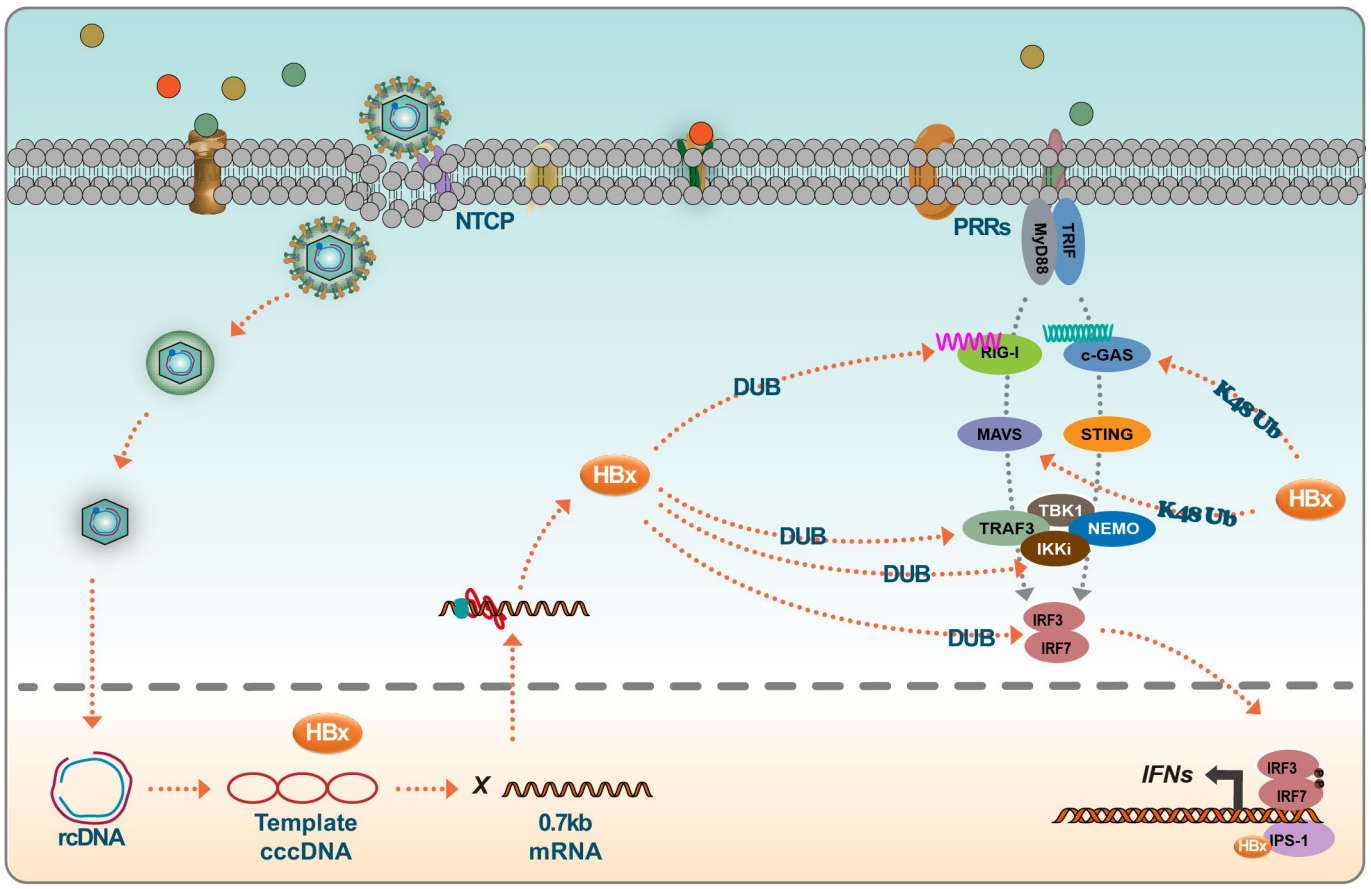
HBx inhibits the IFN signaling. Host cells trigger a series of innate immune responses upon viral infection via pattern recognition receptors (PRRs). Activation of the PRR signaling pathway leads to the activation of several transcription factors, such as IRF3. Viral DNA is recognized by cGAS, which activates STING, which further recruits TBK1 to activate IRF and translocates to the nucleus to initiate IFN production. RIG-I and MDA5 detect RNA and recruit another splicing protein, MAVS (IPS-1), which leads to the activation of TBK1 and phosphorylation of IRFs, thus inducing IFN production. However, interferon production is largely influenced by HBx viral proteins, which, on the one hand, degrade protein molecules such as MAVS and c-GAS by promoting ubiquitination, and on the other hand, HBx itself acts as a deubiquitinating enzyme (DUB), which can target multiple proteins of the signaling pathway. In addition, HBx resolves conjugated IRF7 and binds to CARDIF, TRIF, NEMO, and TBK1, thereby inhibiting the induction of IFN.

Further experimental insights emanate from studies investigating interferon resistance. A model of IFN α-2b-resistant HepG2.2.15 cells was developed through chronic exposure to low doses of IFN α-2b (10–70 IU/mL) for 6–24 weeks. Analyses comparing protein dynamics in the JAK–STAT signaling pathway before and post-development of drug resistance revealed notable perturbations. Specifically, there was a marked decrease in the phosphorylation of STAT1 in the IFNα-resistant HepG2.2.15 cells, concomitant with a down-regulation in the expression of the 2′,5′-oligoadenylate synthetase 1. These findings suggest that disruptions in the JAK–STAT pathway, particularly reduced STAT1 phosphorylation, could support IFN-α treatment resistance observed in HBV patients (Xu et al., 2021) (Figure 3).

### Regulation of ISGs by HBx

IFNs can induce the expression of hundreds of ISGs. It’s interesting to note that, in certain instances, HBV may evade eliciting an innate immune response. This could either be attributed to the failure of PRRs to recognize HBV or, despite the detection by PRRs, HBV actively suppresses IFN production or its downstream signaling (Suslov et al., 2018). Current research has suggested an association between ISGs, HBV, and PEG-IFN α in the context of hepatitis B treatment. While numerous ISGs have been identified over the years, only a subset has had their antiviral activities and mechanisms delineated. The biological significance and antiviral mechanisms of most ISGs remain enigmatic and warrant further exploration.

As previously highlighted, IFNs can be stratified into three distinct categories. Type I and Type III IFNs, upon binding to their respective receptors –IFNAR1 and IFNAR2, orchestrate a signaling cascade. This cascade starts with the proximity-induced phosphorylation of tyrosine kinase 2 (TyK2) and Janus kinase-1 (JAK-1) on the receptors, consequently activating signal transducer and activator of transcription (STAT) factors 1 and 2. The phosphorylated STAT1 and STAT2, in tandem with IFN regulator 9, form a heterotrimer, referred to as interferon-stimulated gene factor 3 (ISGF3). ISGF3 then translocates to the nucleus, engaging with the ISG regulator element (ISRE) to activate ISG transcription. In contrast, the Type II IFN signaling pathway, following the engagement of its receptors IFNGR-1 and IFNGR-2, induces the recruitment and activation of Jak1 and Jak2 kinases. These activated kinases phosphorylate and activate STAT1 and STAT2, leading to their dimerization into the gamma-activated factor (GAF). The GAF dimer then enters the nucleus, associating with gamma-activated sequences (GAS) to stimulate ISG transcription (Schneider et al., 2014).

TRIM14, a tripartite motif-containing (TRIM) family member, plays a crucial role in regulating the interferon signaling pathway. It assists in modulating IFN production, thereby mediating antiviral responses against viral infections. Research has shown that TRIM14 is an ISG in hepatocytes, and its activation is STAT1-dependent. Furthermore, the IFN-I-TRIM14-HBx axis delineates a novel mechanism through which IFN-I impedes viral replication (Tan et al., 2018). In the vast TRIM protein family, TRIM14 stands out for its role in the IFN signaling cascade. It has been shown to recruit USP14 to cleave the lysine 48 (K48)-linked ubiquitin chain at K414 in the cyclic guanosine monophosphate synthase (cGAS) enzyme, thereby blocking the P62-mediated autophagic degradation of cGAS. This process ultimately facilitates the activation of IFN-I signaling (Chen et al., 2016). TRIM14 also possesses LYS63 linkages at LYS-365, facilitating the recruitment of crucial regulators to the MAVS signaling complex, subsequently increasing the innate immune response (Zhou et al., 2014). Furthermore, the interaction between TRIM14’s SPRY domain and the C-terminal of HBx has been shown to block HBx-mediated HBV replication, predominantly by inhibiting the formation of the SMC-HBx-DDB1 complex. Research has unveiled that TRIM5γ associates with HBx-DDB1 in HBV-infected primary human hepatocytes (PHH). This association is predominantly driven by STAT3 rather than STAT1 (Tan et al., 2019). Furthermore, a peptide derived from the Bbox domain has shown potential in promoting HBx degradation and inhibiting HBV replication. At the same time, TRIM25, found to be induced by IFN in an IL-27-dependent manner, has been shown to amplify the IFN signaling pathway, thereby suppressing HBV replication (Tan et al., 2018). Recent advancements in the field have revealed that TRIM21 targets HBx for ubiquitination and proteasomal degradation. This mechanism restricts the degradation of SMC6 mediated by HBx, ultimately inhibiting HBV replication (Song et al., 2021). Another interesting disclosure is the GPD2-mediated recruitment of TRIM28 to degrade HBx, presenting a potential therapeutic avenue for HBV drug development (Liu et al., 2023). TRIM26 also has an established role in HBV inhibition, promoting the degradation of HBx through ubiquitin-mediated mechanisms, and has been identified as a potential marker for predicting response to PegIFNα in chronic hepatitis B patients (Luo et al., 2022). Highlighting the multifaceted role of TRIM proteins, TRIM25 facilitates the degradation of HBx by mediating its ubiquitination at the HBx-k90 locus and augments viral genome replication. Additionally, TRIM25 has been found to enhance the recognition of pgRNA by RIG-I, promoting an increase in IFN production. This dual functionality of TRIM25 provides essential insights into the host-virus interaction dynamics (Song et al., 2023).

**Figure 4:**
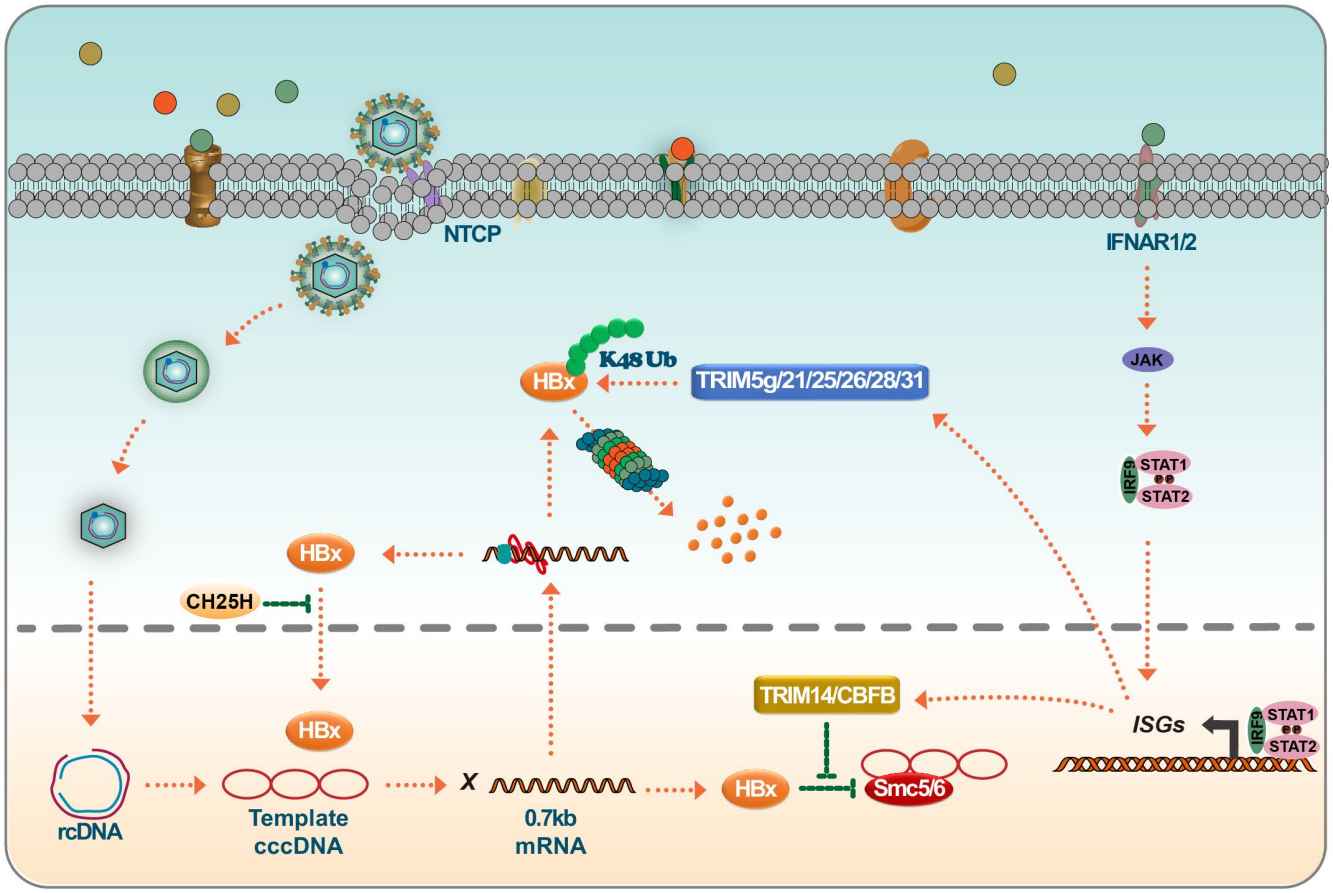
Targeting of HBx by ISGs leads to HBx degradation or loss of function. IFN binds to IFNAR1/2, leading to JAK/STAT activation. Activated ISGF3 trimer or STAT1 dimer binds to ISRE or GAS sequences and promotes the expression of interferon-stimulated genes (ISGs). ISGs such as TRIM14 and CBFβ interact with HBx and prevent HBx-mediated degradation of SMC5/6. On the other hand, many members of the TRIM family, such as TRIM5γ/21/25/26/28/31, degrade HBx viral proteins via K48 chain ubiquitination. Inhibition of a Nrf2 target enzyme (NQO1) with broad cytoprotective functions directly inhibits the production of HBx viral proteins and, interestingly, CH25H, which is involved in cholesterol and lipid metabolism, inhibits nuclear translocation of HBx proteins.

Recent findings suggest that HBV infection mainly induces type III IFN rather than type I or II. Notably, the IL-10 produced in response to type III interferon plays a crucial role in CBFβ synthesis. Investigations have revealed that the HBx associates with the core binding factor (CBF), forming a CBFβ-HBx dimer. This dimer augments CBFβ levels and inhibits the HBx-induced degradation of SMC5/6, ultimately hindering the promotion of HBV replication by HBx (Keasler et al., 2007; Schneider et al., 2014; Song et al., 2021). Further research has demonstrated that IFN-induced expression elevates TRIM26 levels. Subsequently, TRIM26 obstructs HBV replication by promoting the ubiquitination and degradation of HBx. Notably, the SPRY domain of TRIM26 plays a pivotal role in this interaction and degradation process (Luo et al., 2022). Another significant discovery is the role of CH25H, which has been found to effectively curb HBV replication by disrupting the translocation of HBx to the nucleus (Wei et al., 2022). Moreover, ISGs participate in pathways like NF-κB, STAT, Wnt/β-Catenin, ERK, and TGF-β, providing insights into HBx regulation (Figure 4).

## Summary

After HBV infects the host liver, the elicited innate immune response is markedly limited. Over time, HBV has developed numerous evasion techniques to counteract the host cell’s antiviral defense mechanisms. The ultimate goal in treating HBV-infected hepatocytes is to completely clear the virus, ideally by eliminating cccDNA. Current therapeutic strategies can merely control HBV infection or replication rather than achieving complete eradication. Research classifies HBV cures into two types: “functional” and “complete.” A functional cure pertains to the serum clearance of HBsAg, occasionally paired with serum DNA and persistent transcription of inactive cccDNA. In contrast, a complete cure entails the total elimination of cccDNA (Li et al., 2022).

HBx, a non-structural protein and a pivotal regulator of HBV replication, amplifies HBV replication through many mechanisms. These include directly augmenting the transcription of cccDNA, suppressing the expression of antiviral factors, or triggering the degradation of specific host proteins. The increased HBV virulence exacerbates hepatocyte inflammation, leading to hepatitis and potentially progressing to liver fibrosis, cirrhosis, or hepatoma. Besides facilitating HBV replication, HBx independently contributes to hepatocarcinogenesis by activating pathways such as NF-kB, AKT, or p53 or targeting specific oncogenes. HBx also impedes the IFN pathway. Consequently, HBx not only enhances HBV infection, replication, and the progression of HBV-associated diseases but also disrupts the immune response.

This article aims to elucidate the regulatory role of HBx in HBV replication, its impact on the IFN signaling pathway, and its role in HCC metastasis and invasion. This provides a comprehensive theoretical foundation for exploring the role of HBx post-HBV infection. Moreover, this knowledge aids in researching and developing HBx-related small molecule drugs and deepens the understanding of HBx’s role post-HBV infection.

## Author contributions

FW: Writing – original draft. HS: Validation, Writing – original draft. FX: Resources, Validation, Writing – original draft. JX: Validation, Writing – original draft. LW: Validation, Writing – original draft. FY: Validation, Writing – original draft. YZ: Validation, Writing – original draft. GT: Writing – review & editing.
